# ER stress protein AGR2 precedes and is involved in the regulation of pancreatic cancer initiation

**DOI:** 10.1038/onc.2016.459

**Published:** 2016-12-12

**Authors:** L Dumartin, W Alrawashdeh, S M Trabulo, T P Radon, K Steiger, R M Feakins, M P di Magliano, C Heeschen, I Esposito, N R Lemoine, T Crnogorac-Jurcevic

**Affiliations:** 1 Centre for Molecular Oncology, Barts Cancer Institute, Queen Mary University of London, London, UK; 2 Centre for Stem Cells in Cancer & Ageing, Barts Cancer Institute, Queen Mary University of London, London, UK; 3 Institute of Pathology, Technische Universität München, Munich, Germany; 4 Department of Histopathology, Royal London Hospital, London, UK; 5 Department of Surgery, Cell and Developmental Biology, University of Michigan, Ann Arbor, USA; 6 Institute of Pathology, Heinrich-Heine-University of Düsseldorf, Germany

## Abstract

The mechanisms of initiation of pancreatic ductal adenocarcinoma (PDAC) are still largely unknown. In the present study, we analysed the role of anterior gradient-2 (AGR2) in the earliest stages of pancreatic neoplasia. Immunohistochemical analysis of chronic pancreatitis (CP) and peritumoral areas in PDAC tissues showed that AGR2 was present in tubular complexes (TC) and early pancreatic intraepithelial neoplasia (PanINs). Moreover, AGR2 was also found in discrete subpopulations of non-transformed cells neighbouring these pre-neoplastic lesions. In primary cells derived from human patient-derived xenograft (PDX) model, flow-cytometry revealed that AGR2 was overexpressed in pancreatic cancer stem cells (CSC) compared with non-stem cancer cells. In *LSL-Kras*^*G12D*^;*Pdx1-Cre* (*KC*) mouse model Agr2 induction preceded the formation of pre-neoplastic lesions and their development was largely inhibited by Agr2 deletion in engineered *LSL-Kras*^*G12D*^;*Pdx1-Cre*; *Agr2*^*−/−*^ mice. *In vitro*, AGR2 expression was stimulated by tunicamycin-induced endoplasmic reticulum (ER) stress in both *KRAS* wild-type normal pancreas cells, as well as in *KRAS* mutated pancreatic cancer cells and was essential for ER homoeostasis. The unfolded protein response proteins GRP78, ATF6 and XBP1s were found expressed in CP and PDAC peritumoral tissues, but in contrast to AGR2, their expression was switched off during TC and PanIN formation. Real-time PCR and ELISA analyses showed that ER stress induced a pro-inflammatory phenotype in pancreatic normal, cancer and stellate cells. Moreover, AGR2 expression was inducible by paracrine transfer of ER stress and pro-inflammation between different pancreatic cell types. Our findings demonstrate that AGR2 induced in ER-stressed and inflammatory pre-neoplastic pancreas is a potential marker of cancer progenitor cells with an important functional role in PDAC initiation.

## Introduction

Pancreatic ductal adenocarcinoma (PDAC) remains one of the most aggressive and incurable of all malignancies and is projected to become the second leading cause of cancer-related deaths by 2030.^1^ The main reason for this dismal prognosis is the asymptomatic nature of the early stages of the disease resulting in the vast majority of patients to present at an advanced stage of malignancy. Understanding the mechanisms of PDAC initiation is therefore essential for the development of preventive measures, early detection techniques and timely therapeutic intervention.

PDAC and its most common precursor lesions, pancreatic intraepithelial neoplasias (PanINs), are thought to mainly originate from tubular complexes (TC) formed in the centroacinar-acinar compartment through a reprogramming process named acinar-to-ductal metaplasia or through the proliferation of centroacinar cells.^2^ The *KRAS* gene is found mutated (*KRAS*^*G12D*^) almost ubiquitously in PDAC and PanIN lesions^3^ and is considered the key driver oncogene in pancreatic cancer initiation. Preclinical mouse models expressing oncogenic *Kras* under the control of specific pancreatic promoters (for example *Pdx-1*, *Elas*) develop TC, the full spectrum of PanINs and PDAC.^2^

There is growing evidence that *KRAS*-induced carcinogenesis is promoted by cellular stress like chronic inflammation, which plays a crucial role in tumour initiation and progression. Experimentally induced inflammation has been shown to be critical in oncogenic *Kras*-induction of PanINs and PDAC in adult mice and in humans, chronic pancreatitis (CP) has been identified as one of the major risk factors for development of PDAC, and both of these diseases share the presence of TC and PanINs.^4, 5, 6, 7^

Pancreatic (and other) cancer cells frequently experience endoplasmic reticulum (ER) stress resulting from the accumulation of misfolded proteins in the ER due to protein overexpression, oxidative stress, hypoxia, nutrient deprivation and/or calcium depletion. Cells adapt to ER stress by activating the unfolded protein response (UPR) signalling pathway in order to restore ER homoeostasis, but in prolonged or severe stress conditions, cellular signalling switches from pro-survival to ER stress-induced apoptosis.^4^ UPR has been shown to be constitutively active in exocrine pancreas due a high demand for protein synthesis and to be essential for acinar cells homoeostasis.^5^ However, ER stress can induce cellular inflammation^6^ and has been related to the induction of CP.^7, 8^

Anterior gradient 2 (AGR2) is a member of the protein disulphide isomerase family of ER-resident proteins. As such, it is involved in maturation of various proteins; through forming disulphide bonds with mucins, it directly regulates their processing and secretion. Importantly, AGR2 is induced by ER stress, mainly through activation of the ATF6 and IRE1 arms of the UPR,^9^ and participates in the control of ER homoeostasis; AGR2-silenced cells display reduced quality control and folding abilities and are highly sensitive to ER-stress.^10^

AGR2 is also a pro-oncogenic protein that is expressed in various cancers, where it can regulate p53 signalling and induce EGFR ligand amphiregulin to enhance cell survival and spur cancer cell growth (reviewed in ref. 9). In PDAC, AGR2 is found activated downstream of mutant *Kras*^*G12D*^ ^(ref.^ ^11^^)^ and its important role in PDAC development was shown using both orthotopic ^12^ and *Kras*-driven pancreatic cancer mouse model with deletion of *Smad4*, a tumour suppressor gene frequently lost in the late stage (PanIN-3) of pancreatic carcinogenesis.^13^ We showed previously that AGR2 is found, in addition to primary cancer, circulating tumour cells and metastatic lesions, to be invariably induced in patients from the earliest PanIN lesions to PDAC.^14^ Interestingly, AGR2 has recently been described as a potential marker of the cellular origin of oesophageal and cervical cancers.^15, 16^ This has prompted us to analyse the induction and the role of AGR2 in the earliest stages of pancreatic neoplasia and to explore its potential connection with ER stress in PDAC.

Here, we present evidence that in the initial course of PDAC development AGR2 can be induced in pancreatic cells as a response to ER stress and/or oncogenic *KRAS* even before visible neoplasia; we reveal a critical role for AGR2 in the formation of *KRAS*-driven pre-neoplastic lesions, and we demonstrate that ER stress is associated with the development of a pro-inflammatory microenvironment.

## Results

### AGR2 is induced in nontransformed, inflamed and metaplastic exocrine pancreas

**Figure 1:**
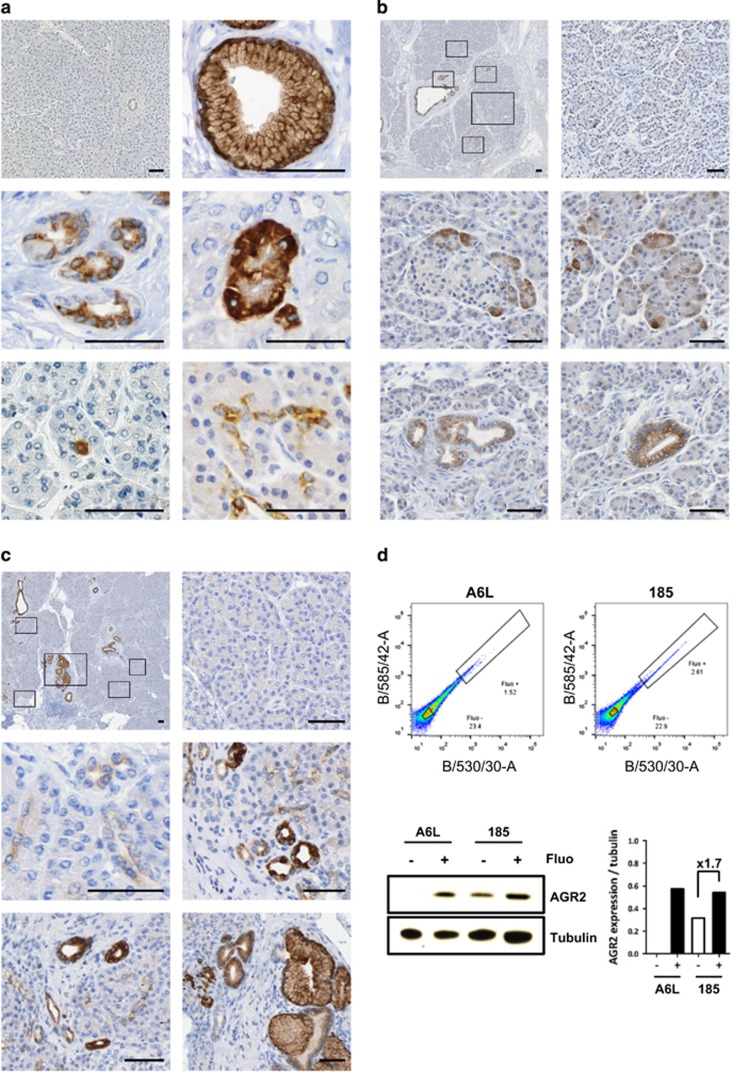
AGR2 expression in human pancreas. (**a**) Immunohistochemical analysis of AGR2 expression in chronic pancreatitis (CP) and PDAC peritumoural tissues. AGR2 protein expression was absent in most of the pancreas (top left) but induced in early PanINs (top right) and tubular complexes, CT (central left), as well as focally induced in phenotypically normal acinar (central right), centroacinar cells (bottom left) and duct/terminal duct (bottom right). (**b**) AGR2 expression in representative peritumoural area. Low magnification of a pancreas tissue (top left) showing area with no AGR2 expression (top right), AGR2 induction in acinar cells (central panels) and AGR2-expressing putative pre-neoplastic lesions, TC (bottom left) and PanIN (bottom right). (**c**) AGR2 expression in representative CP tissue. Low magnification of a pancreas (top left) showing area with no AGR2 expression (top right), moderate AGR2 induction in small and terminal ducts (central left) and strong AGR2 expression in TC and PanINs (central right and bottom panels). Scale bars, 50 μm. (**d**) FACS sorted autofluorescent (Fluo+) and non-autofluorescent (Fluo-) populations of two different PDX-derived primary cell lines (A6L and 185, top panels) were analysed for AGR2 expression by Western blot (bottom left). Densitometry analysis is shown on the right panel.

To examine the induction of AGR2 expression in the early stages of PDAC development, we performed a detailed immunohistochemical analysis in human CP and in the tissue compartment adjacent to cancer, both characterized by the presence of pre-neoplastic lesions (Figure 1; Supplementary Figure S1). While normal pancreas was largely negative (Figure 1a, first panel), AGR2 was found expressed in a similar pattern in peritumoral tissues and CP. We showed that AGR2 was expressed in all PanINs with moderate to high-intensity, displaying a cytoplasmic and membranous pattern (Figure 1a and ref. 14). In addition, AGR2 was also found expressed in TC, as well as in subpopulations of phenotypically normal-appearing acinar, centroacinar and ductal/terminal ductal cells. These phenotypically normal AGR2-positive cells were found in the vicinity or in direct contact to preneoplastic lesions (Figures 1b and c), suggesting that they can be potential precursors to TC and PanINs. For example, peritumoral tissues (Figure 1b) mostly show no AGR2 expression, but AGR2-positive acinar-centroacinar cells were detected in the periphery of pre-neoplastic AGR2-positive lesions; CP tissues (Figure 1c) also show no expression, except for AGR2-positive terminal ducts that were found surrounding TC and PanIN lesions. Phenotypically normal AGR2-positive cells were also detected in PDAC cores (Supplementary Figure S1A).

As tumour-initiating cells are thought to display stem cell-like properties, we explored the expression of AGR2 in pancreatic cancer stem cells using autofluorescence as a cancer stem cell (CSC) marker^17^ for isolation using flow-cytometry (Figure 1d and Supplementary Figure S2). We showed by western blot that AGR2 protein was overexpressed in pancreatic CSC isolated from two primary patient-derived cell lines (A6L and 185) when compared to non-stem cancer cells.

**Figure 2:**
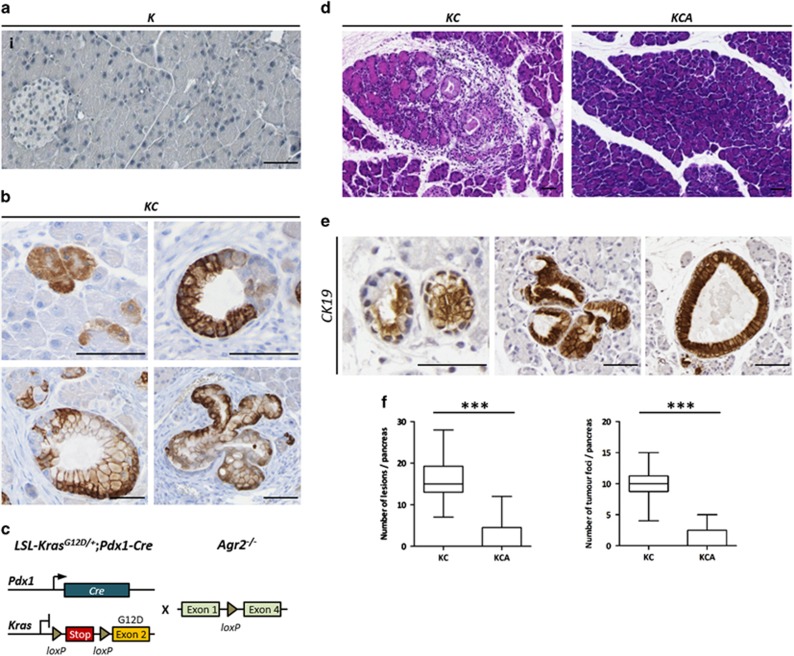
AGR2 loss impairs development of pancreatic pre-neoplastic lesions. (**a**) The absence of AGR2 expression in control *LSL-Kras*^*G12D/+*^ *(K)* mice pancreas. (**b**) AGR2 expression in *LSL-Kras*^*G12D/+*^*;Pdx1-Cre (KC)* mouse model. AGR2 expression in acinar cells in 2 weeks-old *KC* mice (top left) and in tubular complexes (top right), low-grade and high-grade PanINs (left and right bottom panels, respectively) in 4–12 weeks old mice pancreas. Scale bars, 50 μm. (**c**) Genetic make up to activate oncogenic *Kras* in the pancreas using *Cre-loxP* recombination system and subsequent crossing with the *Agr2* knock-out mouse model. (**d**) Representative images of H&E staining of 1 month-old *KC* and *KCA* mice pancreas. (**e**) Cytokeratin 19 (CK19) staining of tubular complexes (left) and early PanINs (central and right panels) in 1 month-old *KC* mice. Scale bars, 50 μm. (**f**) Quantification of the numbers of pre-neoplastic lesions and their foci in *KC* (*n*=13) and *KCA* (*n*=18) mice groups. ***P<0.001.

In mice, AGR2 protein was not expressed in pancreata of control *LSL-Kras*^*G12D/+*^*(K)* animals (Figure 2a). Analysis of germline *Agr2*^*−/−*^ knock-out mouse pancreas showed that *Agr2* deletion does not appear to morphologically alter normal pancreas development (Supplementary Figure S3). In *LSL-Kras*^*G12D/+*^*;Pdx1-Cre (KC)* mouse model (Figure 2b), Agr2 protein started accumulating in phenotypically normal acinar cells in 2 week-old *KC* mice devoid of any apparent pancreatic lesions. In pancreata from 4 to 12 week-old mice, high levels of Agr2 expression were detected in all TC and PanINs, independently of their grade. We have also analysed the *p48-Cre;R26-rtTa-IRES-EGFP;TetO-Kras*^*G12D*^ (*iKras*) mouse model^18^ and observed a similar pattern of expression (representative images are shown on Supplementary Figure S4). Thus, in *Kras*-induced tumourigenesis in mice, Agr2 induction is also an early event that precedes visible neoplasia. All together, these data indicate that pre-neoplastic pancreatic lesions may arise from AGR2-positive progenitor cells.

### Loss of AGR2 impairs early stages of *Kras*-driven pancreatic transformation

To further confirm early AGR2 induction and assess whether AGR2 is implicated in the formation of pre-neoplastic lesions, we knocked-out *Agr2* in *KC* transgenic PDAC mouse model to generate *LSL-Kras*^*G12D/+*^*;Pdx1-Cre;Agr2*^*−/−*^ *(KCA)* mice (Figure 2c). The *KC* mouse develops TC and low-grade PanINs in less than 4 weeks after birth; Figure 2d shows representative pancreas histology of *KC* and *KCA* strains at 1 month. We analysed the effect of *Agr2* loss by quantifying cytokeratin 19-positive TC and low-grade PanINs at that stage (Figure 2e). As shown in Figure 2f, loss of *Agr2* resulted in a significant reduction (*P*<0.001) in both the number of pre-neoplastic lesions (2.23±1.01 vs 15.89±1.19) and their foci (1.15±0.49 vs 9.94±0.65) in *KCA* (*n*=13) compared to *KC* (*n*=18) mouse pancreata, respectively. Moreover, 100% of *KC* mice presented lesions in the pancreas at 1 month whereas >60% of *KCA* mice tissues were histologically normal. Detailed quantification data are presented in Supplementary Table S1. Taken together, these results demonstrated that *Agr2* plays an important functional role in the development of pre-neoplastic lesions during *Kras*-driven tumourigenesis.

### AGR2 is an ER-stress response protein

**Figure 3:**
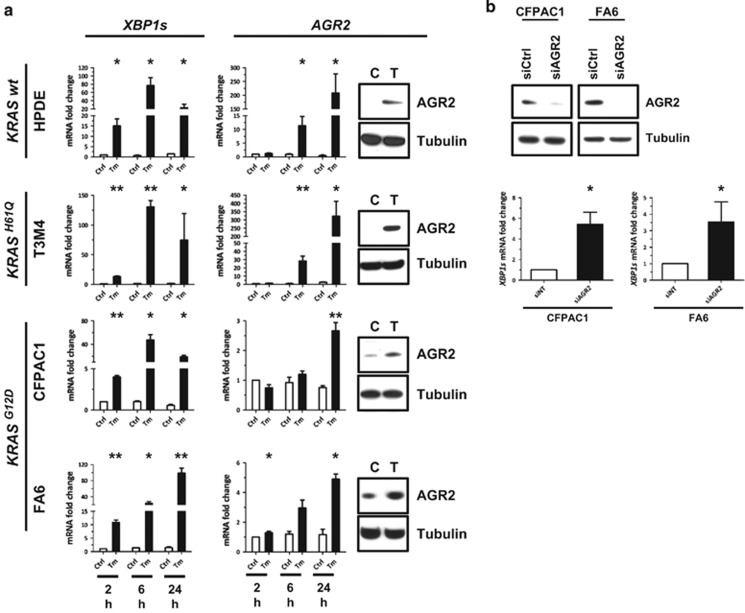
AGR2 is induced by ER stress in pancreatic cells and regulates ER homoeostasis. (**a**) Real-time PCR analysis showed *XBP1s* and *AGR2* gene overexpression in HPDE (*KRAS wt*), T3M4 (*KRAS*^*H61Q*^), and FA6 and CFPAC1 (both *KRAS*^*G12D*^) pancreatic cell lines treated with 5 μM of tunicamycin (Tm) for 2, 6 or 24 h. *S16* was used as a control gene. Western blot analysis (right panels) showed AGR2 protein accumulation after 24 h tunicamycin-treatment; tubulin protein was used as a loading control. (**b**) 72 h after siRNA silencing of *AGR2* in CFPAC1 and FA6 cells (top), overexpression of *XBP1s* gene was observed by real-time PCR (bottom panels). *P<0.05; **P<0.01.

To assess the putative role of ER stress in AGR2 induction in pancreas, four pancreatic cell lines were treated for 2, 6 or 24 h with 5 μM tunicamycin, a well-known ER stress inducer (Figure 3a). We used the *KRAS* wild-type (*wt*) immortalized normal human pancreatic ductal HPDE cells, the *KRAS*^*H61Q*^ mutated PDAC cell line T3M4, and two *KRAS*^*G12D*^ PDAC cell lines: FA6 and CFPAC1. ER stress induction was confirmed by real-time PCR analysis of ER stress marker genes *XBP1s* (Figure 3a), *XBP1*, *GRP78*, *CHOP*, *GADD34* and *ATF4* (Supplementary Figure S5). *XBP1s* appeared to be the most sensitive marker of tunicamycin-induced ER stress. *AGR2* gene expression was shown to be induced in all tested pancreatic cell lines after stress induction. Western blot confirmed the induction of AGR2 protein in all cell lines after 24 h (Figure 3a).

In order to test if AGR2 is functionally involved in ER stress response, we silenced AGR2 in FA6 and CFPAC1 cells that express AGR2 endogenously (Figure 3b). 72 h after silencing of AGR2, real-time PCR showed a significant overexpression of *XBP1s* (bottom panels) and previously tested ER stress markers (data not shown). AGR2 is therefore an ER stress marker in pancreatic cells and is functionally involved in maintenance of ER homoeostasis.

### ER stress and UPR switch in early stages of pancreatic neoplasia

**Figure 4:**
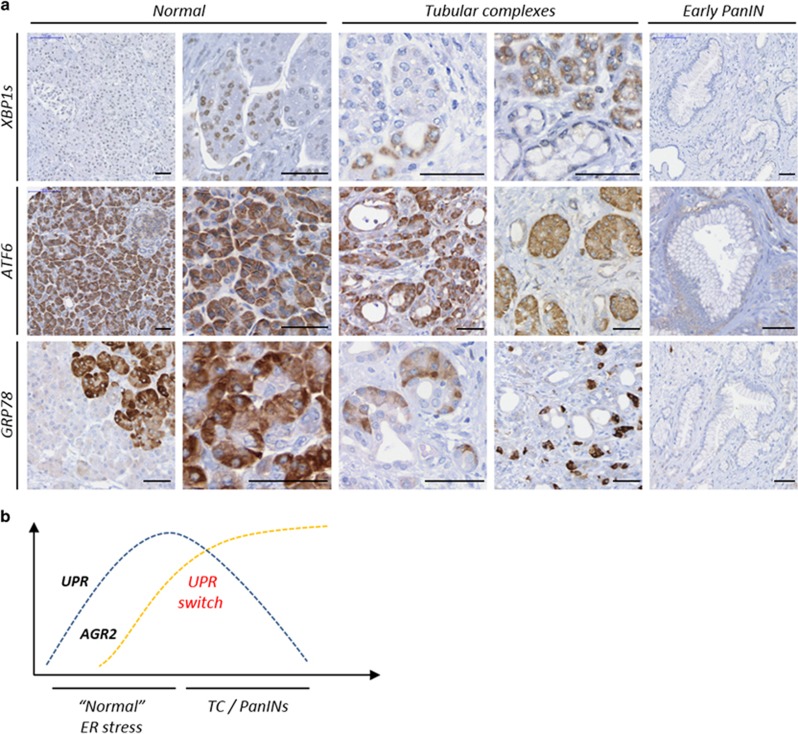
Downregulation of UPR proteins expression during PDAC initiation. (**a**) Immunohistochemical analysis of XBP1s, ATF6 and GRP78 proteins in human chronic pancreatitis. Scale bars, 50 μm. (**b**) Schematic representation of the UPR switch during formation of pancreatic pre-neoplastic lesions. TC, tubular complexes; PanIN, pancreatic intraepithelial neoplasia.

CP is thought to be associated with an abnormal response to ER stress.^7^ To assess the potential role of ER stress response in PDAC initiation, we analysed the expression of ER stress markers and UPR regulator proteins XBP1s, ATF6 and GRP78 in both CP and peritumoural tissues on the same TMAs used to explore AGR2 expression. The three UPR proteins presented similar expression pattern in peritumoral tissues (data not shown) and CP; representative CP images are shown in Figure 4a. XBP1s showed nuclear expression in some acinar and individual ductal cells. In TC, XBP1s showed low to high cytoplasmic expression but PanIN lesions were predominantly negative. ATF6 expression was moderate to strong in normal acinar cells and weak to absent in normal ducts, TC and PanINs. GRP78 expression was heterogeneous in normal acinar cells with weak to strong intensity and was weakly positive or lost in TC and PanINs. Therefore, we confirmed that ER stress is activated in CP and peritumoral regions and revealed that the development of pancreatic pre-neoplastic lesions is associated with the loss of canonical ER stress response proteins. As illustrated in Figure 4b, this UPR switch is characterized by an inverse correlation in the expression of classical UPR proteins and AGR2 during early pre-cancerous lesions development.

### ER stress activates pancreatic stellate cells and links AGR2 to a pro-inflammatory phenotype

**Figure 5:**
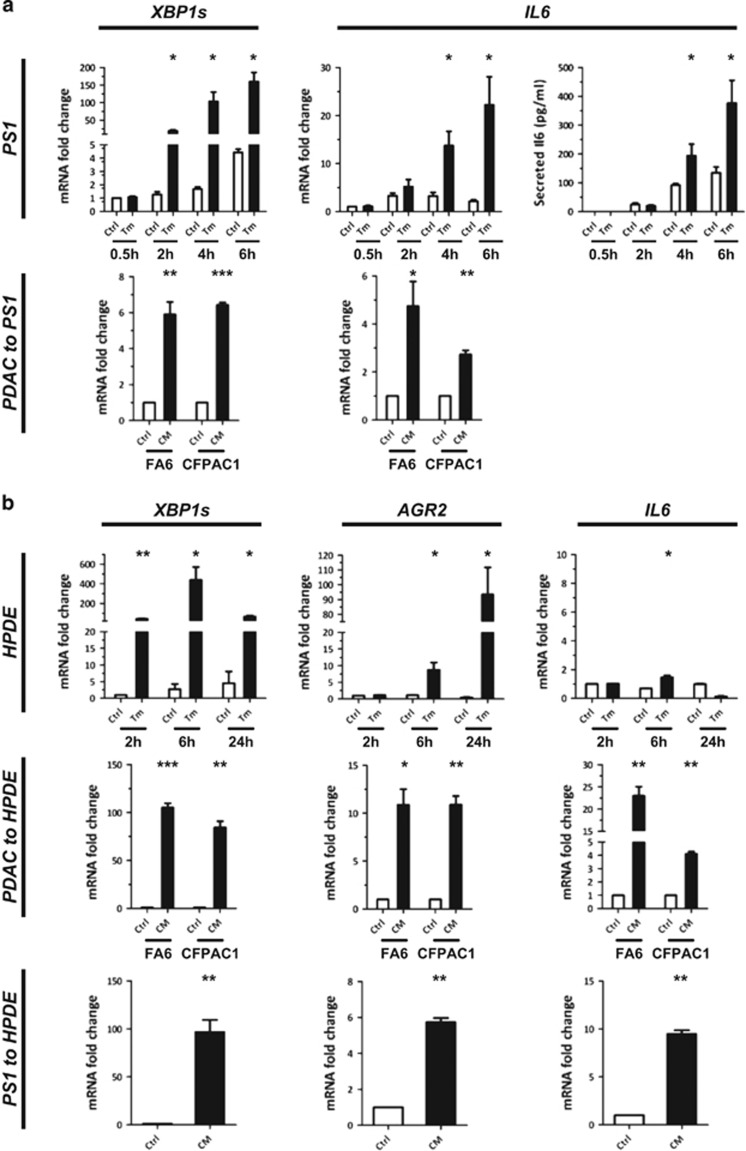
ER-stressed pancreatic cells adopt a pro-inflammatory phenotype. (**a**) ER stress induction analysis in PS1 human pancreatic stellate cells. PS1 cells were treated with 1 μM tunicamycin or vehicle for the indicated times (top panels). *XBP1s* and *IL6* gene expression was analysed by real-time PCR and IL6 protein levels by ELISA analysis of culture supernatants (right panel). PS1 cells were treated with conditioned media from ER-stressed or control pancreatic cancer cells and *XBP1s* and *IL6* gene expression analysed by real-time PCR (bottom panels). (**b**) ER stress induction analysis in human normal pancreatic HPDE cells after treatment with 5 μM Tm (top panels), with conditioned media from ER-stressed or control pancreatic cancer cells (central panels), or from ER-stressed or control PS1 pancreatic stellate cells (bottom panels). *XBP1s*, *AGR2* and *IL6* gene expression was analysed by real-time PCR. Ctrl, control; CM, ER stress-conditioned medium; PC, pancreatic cancer cells; Tm, Tunicamycin. *P<0.05; **P<0.01; ***P<0.001.

Pancreatic stellate cells have been shown to play a critical role in CP and PDAC development which is associated with their acquisition of an inflammatory phenotype.^19^ To further confirm the role of ER stress in PDAC initiation, we tested *in vitro* the effect of ER stress on PS1 pancreatic stellate cells. PS1 cells were treated with 1 μM of tunicamycin for 30 min to 6 h. Real-time PCR analysis of *XBP1s* (Figure 5a, top left panel) and a panel of ER stress markers (Supplementary Figure S6) confirmed the induction of ER stress in PS1 cells. ER stress induction was associated with a significant gene overexpression of the pro-inflammatory cytokine *IL6* and a significant increase in IL6 protein secretion in culture supernatants as measured by ELISA (Figure 5a, top right panels). As ER stress has been shown to be transmissible by cell-to-cell communication,^20^ PS1 cells were then incubated for 6 h with supernatants from ER-stressed or control FA6 and CFPAC1 cell lines (bottom panels). We observed a significant induction of *XBP1s* (Figure 5a, bottom left) and other ER stress markers (Supplementary Figure S6) in PS1 cells treated with ER stress-conditioned media compared to controls. Moreover, ER stress induction was again coupled to an induction of *IL6* gene (Figure 5a, bottom right). Therefore, ER-stressed pancreatic stellate cells adopt a pro-inflammatory phenotype that can be initiated in a cell-autonomous or paracrine manner.

Tunicamycin treatment of HPDE cells led to high and significant increase of *AGR2* expression and moderate, but significant, *IL6* expression (Figure 5b, top). HPDE cells treated with conditioned media from ER-stressed PDAC or PS1 cells (central and bottom panels) also experienced ER stress and showed a significant induction of *AGR2* and *IL6* expression. AGR2 expression and inflammation can thus be induced in pancreatic cells by transmission of ER stress from both cancer and stromal cells.

## Discussion

In this study we demonstrate the importance of AGR2 in the initiation of PDAC. Through combined observations from both human tissues and mouse genetic models, we showed that AGR2 is a marker of pancreatic pre-neoplasia. While we previously showed that AGR2 is induced from the earliest PanINs,^14^ here, we revealed that AGR2 induction precedes PanIN formation as it is also expressed in their putative precursor lesions, tubular complexes. Interestingly, in the close vicinity of these lesions we found subpopulations of phenotypically normal-appearing AGR2-expressing cells that could represent PDAC-initiating cells; this is analogous to the population of AGR2-expressing cells identified as initiating cervical and oesophageal cancers.^15, 16^ This hypothesis is reinforced by the observation of a similar expression pattern in murine models, which are amenable to kinetic analysis, where AGR2 was absent in normal mouse pancreas but was expressed in the pancreas of *KC* mice before any noticeable neoplasia. This early induction further implicates AGR2 as a putative downstream target of mutated *Kras*^*G12D*^signalling, as recently reported.^11^ Similarly in the *Kras;Dicer*^*Het*^ mice, it was shown that the mutated *Kras* may compromise acinar identity prior the induction of ductal genes and morphology, and that it contributes to the upregulation of several genes/pathways even before acinar-to-ductal metaplasia occurs; importantly, *Agr2* was one of these genes.^21^ This not only further supports our mouse data with loss of *Agr2* impairing PanIN development in KC mice, but also mimics our immunohistochemistry data in human tissues.

As we find AGR2 is later expressed in all *Kras*-induced pre-neoplastic lesions and AGR2 knock-out largely inhibited their development, this suggests that pre-neoplastic lesions may originate from AGR2-positive precursors observed in nontransformed pancreas. AGR2 was found induced in different cell types, including acinar, centroacinar cells and terminal ducts, which is in agreement with the multiple potential cellular origin proposed for the development of PDAC.^22, 23, 24, 25, 26, 27, 28^

We analysed the role of AGR2 *in vivo* using the prenatal *KC* model crossed with the germ-line *Agr2* knock-out model. While it was shown previously that heterozygosity for *Agr2* associated with *Smad4* deletion (a late event in PDAC development) could delay PanIN development,^13^ here we demonstrate the importance of *Agr2* in the initiation of *Kras*-driven tumourigenesis early and independently of any late stage mutations. Of note, as *Agr2* knock-out mice tend to develop intestinal complications,^13^ we only conducted a short term *in vivo* experiment to avoid any extra-pancreatic influence. The use of an inducible murine *Agr2* knock-out model and mutant *Kras*^*G12D*^, in a spatial and temporal manner, would permit a more precise analysis of AGR2 functional role in the initiation of PDAC.^29^ However, such a model was not available at the time we were performing our experiments.

AGR2 was previously shown to regulate the proliferation of stem cells and progenitors and their differentiation into multiple cell lineages in mouse stomach ^30^ and to be important for the self-renewal ability of CSC in head and neck squamous cell carcinoma.^31^ Here, we revealed that AGR2 is also overexpressed at the protein level in pancreatic CSC compared with non-stem cancer cells. Taken together, our data indicate that AGR2 plays an important role in PDAC initiation and development, potentially by regulating the maintenance of stemness and progenitor cell differentiation.

**Figure 6:**
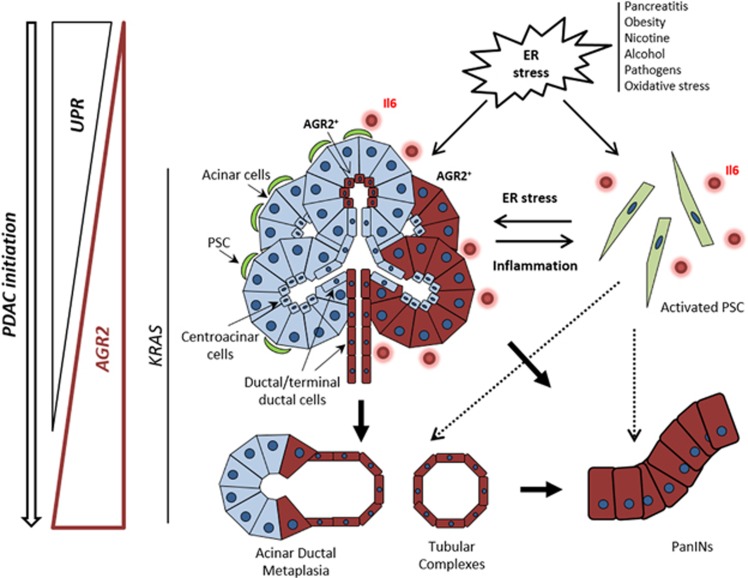
Schematic illustration of AGR2 role and induction in pancreatic cancer initiation. Different conditions and environmental factors (pancreatitis, obesity, smoking, alcohol, pathogens and reactive oxygen species) can cause ER stress in the pancreas. ER stress induces the expression of UPR proteins to restore ER homoeostasis. When UPR is not sufficient to restore homoeostasis in presence of a prolonged or chronic ER stress it can result in inflammatory diseases such as chronic pancreatitis. Paracrine transfer of ER stress between pancreatic cells might further stimulate the development of a pro-inflammatory environment. Occurrence of *KRAS* mutation in such environment is a key driver event for pancreatic cancer initiation. AGR2 expression is both induced by ER stress and *KRAS* mutation and is critical for ER homoeostasis and the early stages of pancreatic carcinogenesis. While AGR2 expression is induced through all the initiating stages of PDAC, UPR proteins appeared to be downregulated during the formation of premalignant lesions. This loss might potentially be directly caused by the occurrence of *KRAS* oncogenic mutation (also stimulating AGR2 expression) and result in protection of cancer-initiating cells from UPR-related apoptosis. Dependency on AGR2 at that stage may therefore reflect its important role in cellular proteostasis during neoplasia.

The great secretory capacity of the exocrine pancreas and its highly developed ER system have led to the hypothesis that ER stress might underlie pancreatic inflammation.^32, 33^ Several known risk factors for pancreatic cancer, such as chronic inflammation, obesity, diabetes, smoking, *etc*. are all associated with ER stress induction^8, 34, 35, 36, 37^ (Figure 6). Interestingly, we show here that AGR2 can be induced in response to ER stress *in vitro* in pancreatic cells independently of their *Kras* status and that AGR2 and ER stress were invariably activated *in vivo* in CP and peritumoural tissues. It is therefore highly likely that, *in vivo*, ER stress-induction of AGR2 can occur independently, and even before, oncogenic *Kras* mutation in the PDAC initiation model. This has recently been demonstrated by Kong *et al*^38^ where, after caerulein-induced pancreatitis, amongst other genes, a ‘wave' of *Agr2* expression was seen – a high increase during regeneration and decrease during refinement phases in wt mice; in *Kras-*mutated mice after caerulein challenge, *Agr2* upregulation was additionally seen during inflammatory phase, and was pronounced during the refinement phase, which now does not progress to resolution, but rather to acinar-to-ductal metaplasia, PanIN and PDAC development. Therefore, it is likely that by enhancing ER folding capacity, AGR2 allows the pre-cancerous cells to cope with an increased protein demand both before and or/after oncogenic mutations. The fine-tuning and the correlation of AGR2 expression and the levels of KRAS activity in human system however, still remain to be further established.

Interestingly, in contrast to the induction of AGR2, the expression of three UPR proteins XBP1s, ATF6 and GRP78, essential for cellular proteostasis, was lost during the development of premalignant lesions (the ATF4/CHOP UPR branch of the ER stress could not be assessed due to suboptimal quality of IHC data). This early switch might be essential for cancer initiating cells to survive ER stress whilst avoiding UPR-related apoptosis. The antiapoptotic role of AGR2, at least *in vitro*, was recently demonstrated through the direct association of its homodimer with GRP78.^39^

The demonstrated *in vivo* dependency on AGR2 for the formation of premalignant lesions is likely due to its important role in cellular proteostasis during cell transformation, and induction of AGR2 in pancreas might therefore be a marker of pathologic response to proteotoxic stress.

Finally, we also revealed that ER stress-induction of AGR2 is associated with the acquisition of a pro-inflammatory phenotype and that ER stress could be the mediator of an intercellular communication between both epithelial and stromal cells (Figure 6) as shown here for pancreatic stellate cells and previously for macrophages.^20^ The pronounced induction of AGR2 by the conditioned media of ER stressed cells could potentially be explained by the deregulation of a large panel of secreted proteins (in addition to IL6), some of which could represent potential paracrine candidates. To this end we have tested if IL6 could exert such an effect, however both siRNA silencing of IL6 or the treatment of cells with recombinant IL6 protein, did not affect AGR2 expression levels (data not shown). It is thus plausible that other paracrine mediators, including inflammatory mediators such as reactive oxygen species (ROS), are involved in the intercellular communication between ER stress and AGR2 expression; however, this hypothesis remains to be addressed further.

Based on the presented findings, it is tempting to speculate that the crosstalk between the stressed epithelial and stromal cells might also contribute *in vivo* to the initiation and progression of PDAC by stimulating the development of an inflammatory environment as well as promoting field cancerization.

In summary, our study sheds new light on PDAC initiation and development and reveals the induction of the pro-oncogenic AGR2 protein in pancreas before manifested neoplasia. Acquisition of resistance against UPR-induced cell death during chronic ER stress conditions might be an early and widespread mechanism in PDAC development, and targeting AGR2 could be a promising strategy to reverse this acquired survival advantage and increase cancer cell vulnerability. In this way, targeting ER stress resistance in combination with ER stress-inducing drugs, like IPI-504 ^40^ or bortezomib,^41, 42^ represents a promising preventive or therapeutic strategy against aggressive *KRAS*-driven tumours like pancreatic cancer.

## Materials and methods

### Cells and tissues

The immortalized human pancreatic duct epithelial cell line HPDE was obtained from Dr Ming-Sound Tsao (University of Toronto); the immortalized normal human pancreatic stellate cell line PS1 was obtained from Prof. Hemant Kocher (Barts Cancer Institute, London); both cell lines were grown as described previously.^43, 44^ The remaining cell lines were obtained from Cancer Research UK Cell Services and cultured in Dulbecco's Modified Eagle Medium, DMEM, (Invitrogen, Paisley, UK) supplemented with 10% heat-inactivated fetal calf serum (Autogen Bioclear, Wiltshire, UK). The identity of cell lines was verified by STR profiling. PDAC patient-derived xenografts (PDXs) (referred as anonymous patient numbers: 185 and A6L) were processed as previously described.^45^ Human tissue microarrays (TMAs) comprising acinar-to-ductal metaplasia, TC, PanIN, CP and PDAC lesions used for immunohistochemcal analyses were constructed as described previously.^2^

### RNA extraction and semi-quantitative real-time PCR

Total RNA was extracted using RNAqueous RNA extraction kit (Ambion, Warrington, UK). complementaryDNA (cDNA) was synthesized from 1 μg of total RNA with Quantitect Reverse Transcription kit (Qiagen, Crawley, UK). Real-time PCR was performed using a 7500 Real Time PCR System (Applied Biosystems, Warrington, UK) using SYBR Green dye (Qiagen). Primer sequences are listed in Supplementary Table S2. All samples were analysed in three independent experiments. Relative expression changes were presented after normalization to the human ribosomal *S16* gene.

Primers and real-time PCR for pluripotency genes (Nanog, Klf4, Sox2, Oct3/4 and Nodal) have been described in ref. 17

### Gene silencing

Cells were seeded at 2 × 10^5^ cells per well in a 6-well plate and transfected with 50 nM of siGENOME ON-TARGETplus SMARTpool siRNA specific for human AGR2 or siGENOME Non-Targeting siRNA pool #2 (Dharmacon, Chicago, USA) using INTERFERin (PeqLab, Fareham, UK) according to manufacturer's instructions.

### Flow cytometry and cell sorting

Cells were harvested by trypsinisation and resuspended in PBS; 3% FBS (v/v); 1 mM EDTA before sorting with a BD FACSAria II Cell sorter (BD Biosciences, Oxford, UK). Autofluorescent cells were excited with a 488 nm blue laser and selected as the intersection with filters 530/30 and 585/42. Some non-autofluorescent cells are lost during sorting due to the gate settings that maintain an appropriate distance between autofluorescent and non-autofluorescent cells. However, such settings are required to ensure high purity. DAPI staining was used for exclusion of dead cells.

### Western blotting

Cell lysis was performed using NP40 buffer (1% NP40, 50 mM Tris pH7.4, 150 mM NaCl) with protease inhibitors (Roche Diagnostics, West Sussex, UK). 25 μg of protein lysate were analysed by SDS–polyacrylamide gel electrophoresis (SDS–PAGE), as previously described.^46^ Primary antibodies used were rabbit anti-AGR2 1:250 (Novus Biologicals, Cambridge, UK) and anti-tubulin 1:500 (Abcam, Cambridge, UK).

### Immunohistochemistry

Immunostaining was performed on 4 μm-thick paraffin sections using rabbit anti-AGR2 antibody (Abcam, 1:30), anti-XBP1s (Monoclonal Antibodies Unit, CNIO, Madrid, Spain 1:100), anti-ATF6 (Abcam, 1:8000), anti-GRP78 (Cell Signalling Technology, Hitchin, UK, 1:200) with DABMap kit, following protocols for the Ventana Discovery System (Illkirch, France). Counterstaining was performed with haematoxylin.

### Endoplasmic reticulum stress assays

Cells were incubated with 1 or 5 μM tunicamycin (Sigma-Aldrich, Gillingham, Dorset, UK) in complete media for 2, 6 or 24 h. To obtain ER stress-conditioned medium, tumour cells were cultured in 5 μM tunicamycin or vehicle (100% DMSO) for 2 h. Cells were washed twice with PBS and then incubated in fresh medium for 16 h. Conditioned medium was centrifuged for 10 min at 2,000 *g* and then passed through a 0.22 μm filter (EMD Millipore, Hertfordshire, UK).

### Mouse experiments

*LSL-Kras*^*G12D/+*^ *(K)* and *Pdx1-Cre (C)* mice provided by Prof. David Tuveson (Cold Spring Harbor Laboratory) were interbred to generate *LSL-Kras*^*G12D/+*^*;Pdx1-Cre (KC)* mice. In the KC model, the expression of activated *Kras* is targeted to the mouse pancreas using a conditional *LSL-Kras*^*G12D/+*^ allele activated by Cre-mediated recombination, with Cre under the control of the pancreatic and duodenal homeobox1 promoter (*Pdx1*). Germline *Agr2*^*−/−*^ mice on a C57Bl/6 background provided by Prof. David Erle (University of California, San Francisco) were interbred with *KC* mice to generate *LSL-Kras*^*G12D/+*^*;Pdx1-Cre;Agr2*^*−/−*^ *(KCA)* mouse strain. Wild-type (*wt*) mice were purchased from Charles River Laboratory. For the quantification of pancreatic lesions, haematoxylin and eosin (H&E) or CK19 stained slides were analysed after scanning using a Pannoramic 250 High Throughput Scanner (3Dhistech, Budapest, Hungary). All procedures were approved by the QMUL animal ethics committee and were executed in accordance with UK Home Office regulations.

### Statistical analysis

The statistical analysis was performed with the two-tailed paired Student's *t*-test using GraphPad Prism software; a *P* value of <0.05 was chosen as statistically significant. Column bar graphs show mean and s.e.m. of each data set.

## Abbreviations

AGR2: Anterior gradient-2
CSC: Cancer stem cell
CP: Chronic pancreatitis
ER: Endoplasmic reticulum
KC: *LSL-Kras*^*G12D*^, *Pdx1-Cre*
TC: Tubular complexes
PDAC: Pancreatic ductal adenocarcinoma
PanIN: Pancreatic intraepithelial neoplasia.

## References

[bib1] Rahib L, Smith BD, Aizenberg R, Rosenzweig AB, Fleshman JM, Matrisian LM. Projecting cancer incidence and deaths to 2030: the unexpected burden of thyroid, liver, and pancreas cancers in the United States. Cancer Res 2014; 74: 2913–2921. 24840647 10.1158/0008-5472.CAN-14-0155

[bib2] Aichler M, Seiler C, Tost M, Siveke J, Mazur PK, Da Silva-Buttkus P et al. Origin of pancreatic ductal adenocarcinoma from atypical flat lesions: a comparative study in transgenic mice and human tissues. J Pathol 2012; 226: 723–734. 21984419 10.1002/path.3017

[bib3] Kanda M, Matthaei H, Wu J, Hong SM, Yu J, Borges M et al. Presence of somatic mutations in most early-stage pancreatic intraepithelial neoplasia. Gastroenterology 2012; 142: 730–733 e9. 22226782 10.1053/j.gastro.2011.12.042 PMC3321090

[bib4] Hetz C, Chevet E, Oakes SA. Proteostasis control by the unfolded protein response. Nat Cell Biol 2015; 17: 829–838. 26123108 10.1038/ncb3184 PMC5546321

[bib5] Hess DA, Humphrey SE, Ishibashi J, Damsz B, Lee AH, Glimcher LH et al. Extensive pancreas regeneration following acinar-specific disruption of Xbp1 in mice. Gastroenterology 2011; 141: 1463–1472. 21704586 10.1053/j.gastro.2011.06.045 PMC3186847

[bib6] Garg AD, Kaczmarek A, Krysko O, Vandenabeele P, Krysko DV, Agostinis P. ER stress-induced inflammation: does it aid or impede disease progression? Trends Mol Med 2012; 18: 589–598. 22883813 10.1016/j.molmed.2012.06.010

[bib7] Lugea A, Tischler D, Nguyen J, Gong J, Gukovsky I, French SW et al. Adaptive unfolded protein response attenuates alcohol-induced pancreatic damage. Gastroenterology 2011; 140: 987–997. 21111739 10.1053/j.gastro.2010.11.038 PMC3057335

[bib8] Sah RP, Garg SK, Dixit AK, Dudeja V, Dawra RK, Saluja AK. Endoplasmic reticulum stress is chronically activated in chronic pancreatitis. J Biol Chem 2014; 289: 27551–27561. 25077966 10.1074/jbc.M113.528174 PMC4183795

[bib9] Chevet E, Fessart D, Delom F, Mulot A, Vojtesek B, Hrstka R et al. Emerging roles for the pro-oncogenic anterior gradient-2 in cancer development. Oncogene 2013; 32: 2499–2509. 22945652 10.1038/onc.2012.346

[bib10] Higa A, Mulot A, Delom F, Bouchecareilh M, Nguyen DT, Boismenu D et al. Role of pro-oncogenic protein disulfide isomerase (PDI) family member anterior gradient 2 (AGR2) in the control of endoplasmic reticulum homeostasis. J Biol Chem 2011; 286: 44855–44868. 22025610 10.1074/jbc.M111.275529 PMC3248018

[bib11] Boj SF, Hwang CI, Baker LA, Chio II, Engle DD, Corbo V et al. Organoid models of human and mouse ductal pancreatic cancer. Cell 2015; 160: 324–338. 25557080 10.1016/j.cell.2014.12.021 PMC4334572

[bib12] Ramachandran V, Arumugam T, Wang H, Logsdon CD. Anterior gradient 2 is expressed and secreted during the development of pancreatic cancer and promotes cancer cell survival. Cancer Res 2008; 68: 7811–7818. 18829536 10.1158/0008-5472.CAN-08-1320 PMC4429896

[bib13] Norris AM, Gore A, Balboni A, Young A, Longnecker DS, Korc M. AGR2 is a SMAD4-suppressible gene that modulates MUC1 levels and promotes the initiation and progression of pancreatic intraepithelial neoplasia. Oncogene 2013; 32: 3867–3876. 22945649 10.1038/onc.2012.394 PMC3515713

[bib14] Dumartin L, Whiteman HJ, Weeks ME, Hariharan D, Dmitrovic B, Iacobuzio-Donahue CA et al. AGR2 is a novel surface antigen that promotes the dissemination of pancreatic cancer cells through regulation of cathepsins B and D. Cancer Res 2011; 71: 7091–7102. 21948970 10.1158/0008-5472.CAN-11-1367 PMC3541941

[bib15] Wang X, Ouyang H, Yamamoto Y, Kumar PA, Wei TS, Dagher R et al. Residual embryonic cells as precursors of a Barrett's-like metaplasia. Cell 2011; 145: 1023–1035. 21703447 10.1016/j.cell.2011.05.026 PMC3125107

[bib16] Herfs M, Yamamoto Y, Laury A, Wang X, Nucci MR, McLaughlin-Drubin ME et al. A discrete population of squamocolumnar junction cells implicated in the pathogenesis of cervical cancer. Proc Natl Acad Sci USA 2012; 109: 10516–10521. 22689991 10.1073/pnas.1202684109 PMC3387104

[bib17] Miranda-Lorenzo I, Dorado J, Lonardo E, Alcala S, Serrano AG, Clausell-Tormos J et al. Intracellular autofluorescence: a biomarker for epithelial cancer stem cells. Nat Methods 2014; 11: 1161–1169. 25262208 10.1038/nmeth.3112

[bib18] Collins MA, Bednar F, Zhang Y, Brisset JC, Galban S, Galban CJ et al. Oncogenic Kras is required for both the initiation and maintenance of pancreatic cancer in mice. J Clin Investig 2012; 122: 639–653. 22232209 10.1172/JCI59227 PMC3266788

[bib19] Sherman MH, Yu RT, Engle DD, Ding N, Atkins AR, Tiriac H et al. Vitamin D receptor-mediated stromal reprogramming suppresses pancreatitis and enhances pancreatic cancer therapy. Cell 2014; 159: 80–93. 25259922 10.1016/j.cell.2014.08.007 PMC4177038

[bib20] Mahadevan NR, Rodvold J, Sepulveda H, Rossi S, Drew AF, Zanetti M. Transmission of endoplasmic reticulum stress and pro-inflammation from tumor cells to myeloid cells. Proc Natl Acad Sci USA 2011; 108: 6561–6566. 21464300 10.1073/pnas.1008942108 PMC3081038

[bib21] Morris JPt, Greer R, Russ HA, von Figura G, Kim GE, Busch A et al. Dicer regulates differentiation and viability during mouse pancreatic cancer initiation. PLOS One 2014; 9: e95486. 24788257 10.1371/journal.pone.0095486 PMC4006805

[bib22] Gidekel Friedlander SY, Chu GC, Snyder EL, Girnius N, Dibelius G, Crowley D et al. Context-dependent transformation of adult pancreatic cells by oncogenic K-Ras. Cancer Cell 2009; 16: 379–389. 19878870 10.1016/j.ccr.2009.09.027 PMC3048064

[bib23] Guerra C, Mijimolle N, Dhawahir A, Dubus P, Barradas M, Serrano M et al. Tumor induction by an endogenous K-ras oncogene is highly dependent on cellular context. Cancer Cell 2003; 4: 111–120. 12957286 10.1016/s1535-6108(03)00191-0

[bib24] Habbe N, Shi G, Meguid RA, Fendrich V, Esni F, Chen H et al. Spontaneous induction of murine pancreatic intraepithelial neoplasia (mPanIN) by acinar cell targeting of oncogenic Kras in adult mice. Proc Natl Acad Sci USA 2008; 105: 18913–18918. 19028870 10.1073/pnas.0810097105 PMC2596215

[bib25] Kopp JL, von Figura G, Mayes E, Liu FF, Dubois CL, JPt Morris et al. Identification of Sox9-dependent acinar-to-ductal reprogramming as the principal mechanism for initiation of pancreatic ductal adenocarcinoma. Cancer Cell 2012; 22: 737–750. 23201164 10.1016/j.ccr.2012.10.025 PMC3568632

[bib26] Pylayeva-Gupta Y, Lee KE, Hajdu CH, Miller G, Bar-Sagi D. Oncogenic Kras-induced GM-CSF production promotes the development of pancreatic neoplasia. Cancer Cell 2012; 21: 836–847. 22698407 10.1016/j.ccr.2012.04.024 PMC3721510

[bib27] von Figura G, Fukuda A, Roy N, Liku ME, Morris Iv JP, Kim GE et al. The chromatin regulator Brg1 suppresses formation of intraductal papillary mucinous neoplasm and pancreatic ductal adenocarcinoma. Nat Cell Biol 2014; 16: 255–267. 24561622 10.1038/ncb2916 PMC4684081

[bib28] Roy N, Malik S, Villanueva KE, Urano A, Lu X, Von Figura G et al. Brg1 promotes both tumor-suppressive and oncogenic activities at distinct stages of pancreatic cancer formation. Genes Dev 2015; 29: 658–671. 25792600 10.1101/gad.256628.114 PMC4378197

[bib29] Collins MA, Brisset JC, Zhang Y, Bednar F, Pierre J, Heist KA et al. Metastatic pancreatic cancer is dependent on oncogenic Kras in mice. PLOS One 2012; 7: e49707. 23226501 10.1371/journal.pone.0049707 PMC3513322

[bib30] Gupta A, Wodziak D, Tun M, Bouley DM, Lowe AW. Loss of anterior gradient 2 (Agr2) expression results in hyperplasia and defective lineage maturation in the murine stomach. J Biol Chem 2013; 288: 4321–4333. 23209296 10.1074/jbc.M112.433086 PMC3567683

[bib31] Ma SR, Wang WM, Huang CF, Zhang WF, Sun ZJ. Anterior gradient protein 2 expression in high grade head and neck squamous cell carcinoma correlated with cancer stem cell and epithelial mesenchymal transition. Oncotarget 2015; 6: 8807–8821. 25871396 10.18632/oncotarget.3556 PMC4496185

[bib32] Pandol SJ, Gorelick FS, Lugea A. Environmental and genetic stressors and the unfolded protein response in exocrine pancreatic function - a hypothesis. Front Physiol 2011; 2: 8. 21483727 10.3389/fphys.2011.00008 PMC3070477

[bib33] Pandol SJ, Gorelick FS, Gerloff A, Lugea A. Alcohol abuse, endoplasmic reticulum stress and pancreatitis. Digest Dis 2010; 28: 776–782. 10.1159/000327212 PMC3211518 21525762

[bib34] Ozcan U, Cao Q, Yilmaz E, Lee AH, Iwakoshi NN, Ozdelen E et al. Endoplasmic reticulum stress links obesity, insulin action, and type 2 diabetes. Science 2004; 306: 457–461. 15486293 10.1126/science.1103160

[bib35] Kawasaki N, Asada R, Saito A, Kanemoto S, Imaizumi K. Obesity-induced endoplasmic reticulum stress causes chronic inflammation in adipose tissue. Sci Rep 2012; 2: 799. 23150771 10.1038/srep00799 PMC3495279

[bib36] Jorgensen E, Stinson A, Shan L, Yang J, Gietl D, Albino AP. Cigarette smoke induces endoplasmic reticulum stress and the unfolded protein response in normal and malignant human lung cells. BMC Cancer 2008; 8: 229. 18694499 10.1186/1471-2407-8-229 PMC2527015

[bib37] Celli J, Tsolis RM. Bacteria, the endoplasmic reticulum and the unfolded protein response: friends or foes? Nat Rev Microbiol 2015; 13: 71–82. 25534809 10.1038/nrmicro3393 PMC4447104

[bib38] Kong B, Bruns P, Behler NA, Chang L, Schlitter AM, Cao J et al. Dynamic landscape of pancreatic carcinogenesis reveals early molecular networks of malignancy. Gut 2016, e-pub ahead of print 19 September 2016 doi:10.1136/gutjnl-2015-310913. 10.1136/gutjnl-2015-310913 27646934

[bib39] Ryu J, Park SG, Lee PY, Cho S, Lee DH, Kim GH et al. Dimerization of pro-oncogenic protein Anterior Gradient 2 is required for the interaction with BiP/GRP78. Biochem Biophys Res Commun 2013; 430: 610–615. 23220234 10.1016/j.bbrc.2012.11.105

[bib40] De Raedt T, Walton Z, Yecies JL, Li D, Chen Y, Malone CF et al. Exploiting cancer cell vulnerabilities to develop a combination therapy for ras-driven tumors. Cancer Cell 2011; 20: 400–413. 21907929 10.1016/j.ccr.2011.08.014 PMC3233475

[bib41] Nawrocki ST, Carew JS, Pino MS, Highshaw RA, Dunner K Jr, Huang P et al. Bortezomib sensitizes pancreatic cancer cells to endoplasmic reticulum stress-mediated apoptosis. Cancer Res 2005; 65: 11658–11666. 16357177 10.1158/0008-5472.CAN-05-2370

[bib42] Nawrocki ST, Carew JS, Dunner K Jr, Boise LH, Chiao PJ, Huang P et al. Bortezomib inhibits PKR-like endoplasmic reticulum (ER) kinase and induces apoptosis via ER stress in human pancreatic cancer cells. Cancer Res 2005; 65: 11510–11519. 16357160 10.1158/0008-5472.CAN-05-2394

[bib43] Liu N, Furukawa T, Kobari M, Tsao MS. Comparative phenotypic studies of duct epithelial cell lines derived from normal human pancreas and pancreatic carcinoma. Am J Pathol 1998; 153: 263–269. 9665487 10.1016/S0002-9440(10)65567-8 PMC1852927

[bib44] Froeling FE, Feig C, Chelala C, Dobson R, Mein CE, Tuveson DA et al. Retinoic acid-induced pancreatic stellate cell quiescence reduces paracrine Wnt-beta-catenin signaling to slow tumor progression. Gastroenterology 2011; 141: e1–14. 10.1053/j.gastro.2011.06.047 21704588

[bib45] Mueller MT, Hermann PC, Witthauer J, Rubio-Viqueira B, Leicht SF, Huber S et al. Combined targeted treatment to eliminate tumorigenic cancer stem cells in human pancreatic cancer. Gastroenterology 2009; 137: 1102–1113. 19501590 10.1053/j.gastro.2009.05.053

[bib46] Whiteman HJ, Weeks ME, Dowen SE, Barry S, Timms JF, Lemoine NR et al. The role of S100P in the invasion of pancreatic cancer cells is mediated through cytoskeletal changes and regulation of cathepsin D. Cancer Res 2007; 67: 8633–8642. 17875703 10.1158/0008-5472.CAN-07-0545

